# Preventing lives affected by hemophilia: A mixed methods study of the views of adults with hemophilia and their families toward genetic screening

**DOI:** 10.1002/mgg3.618

**Published:** 2019-03-05

**Authors:** Felicity K. Boardman, Rachel Hale, Raksha Gohel, Philip J. Young

**Affiliations:** ^1^ Warwick Medical School University of Warwick Coventry UK; ^2^ School of Life Sciences University of Warwick Coventry UK

**Keywords:** attitudes, carrier, hemophilia, mixed methods, screening, UK

## Abstract

**Background:**

Genomic sequencing technologies have made the possibility of population screening for whole panels of genetic disorders more feasible than ever before. As one of the most common single gene disorders affecting the UK population, hemophilia is an attractive candidate to include on such screening panels. However, very little is known about views toward genetic screening amongst people with hemophilia or their family members, despite the potential for a wide range of impacts on them.

**Methods:**

Twenty‐two in‐depth qualitative interviews were undertaken to explore the views of adults with hemophilia and their family members, recruited through the Haemophilia Society UK. These interviews were used to develop a survey, the Haemophilia Screening Survey (UK), which was distributed in paper and online format through the support group, receiving 327 returns between January and June 2018.

**Results:**

Fifty‐seven per cent of the sample supported preconception carrier screening of the population for hemophilia, and 59% supported prenatal carrier screening. Key reasons for support included a desire to reduce pregnancy terminations and increase awareness of hemophilia. Despite support for screening however, 90% of the sample disagreed with pregnancy terminations for hemophilia.

**Conclusions:**

Families and adults living with hemophilia are more supportive of screening for information and preparation purposes than to prevent boys with hemophilia from being born. A distinction was made between preventing the disease and preventing the lives of people with it, with support shown for the use of screening to achieve the former, but not at the expense of the latter.

## 1 INTRODUCTION

As the capacities of genomic technologies continue to expand and become more efficient, genomic information is more accessible to members of the public than ever before. Within the UK, the Genomics England initiative, the 100,000 Genomes Project, highlights the high position of population genomics on the public agenda (Davies, 2016) and suggests a future in which population carrier screening using genomic sequencing techniques (potentially also accompanied by genome editing) are an accepted and widely used component of mainstream reproductive healthcare. Supporters of expansive carrier screening for rare genetic diseases have highlighted the significant risk that these disorders collectively pose to the public's health. Whilst individually rare, their combined incidence is comparable to that of Down Syndrome—a condition routinely screened for across the globe (Archibald et al., 2017). Furthermore, it has been argued that the introduction of population carrier screening would expand the reproductive options of carrier parents (Human Genetics Commission, 2011). For example, it would allow them the option of avoiding the birth of an affected child altogether. Indeed, without pre‐conception or prenatal screening, the birth of an affected child is typically the most common route by which carrier parents discover their status. When preconception carrier screening is implemented, however, carrier parents may choose to access other technologies, such as pre‐implantation genetic diagnosis for use in their very first pregnancy to ensure the birth of an unaffected embryo. In addition, the potential for negative health implications associated with carrier status (e.g., in relation to hemophilia, thalassemia, sickle cell, and fragile x syndrome) are also being increasingly recognized, suggesting that carrier screening has wider health implications that go beyond the domain or reproduction (Abdul‐Kadir, Davies, Halimeh, & Chi, 2013). Despite these potential benefits, however, the acceptability of population carrier screens to both the general public, and, more specifically the communities already living with genetic diseases, has been relatively under‐explored, although this body of literature is emerging (Archibald et al., 2013; Henneman et al., 2001; McClaren, Delatycki, Collins, Metcalfe, & Aitken, 2008; Roadhouse et al., 2018). This under‐representation of families living with genetic diseases in debates around genetic screening and selective reproduction is particularly striking as genetic screening would likely have implications for such families beyond those anticipated for the general public (Boardman & Hale, 2018). Such implications may include an increase in social stigmatization of those living with screened‐for conditions (as the public profile of the disorder shifts to a “preventable” disease), the potential for reductions in funding for research into treatments and the loss of peer‐to‐peer support as the incidence of a condition declines over time (Nuffield Council on Bioethics, 2018).

Previous research on attitudes toward screening and selective reproduction has been somewhat contradictory. Some studies have revealed widespread support for selective reproduction amongst affected families and adults (e.g., Chen & Schiffman, 2000; Conway, Allenby, & Pond, 1994; Janssens et al., 2016; Potrata, McKibbin, Lim, & Hewison, 2014), whilst more recent research points to ambivalence and conflict (Maxwell et al., 2011), and sometimes outright rejection of the notion of screening on principle (Barter, Hastings, Williams, & Huws, 2016; Boardman & Hale, 2018; Roadhouse et al., 2018). Concerns have been expressed about the loss of (potentially) high quality life in spite of genetic disease, the implied implicit judgement on the value of life with disability and disapproval of the redirection of resources away from social and environmental barrier removal and toward the medical elimination of the condition (Boardman & Hale, 2018; Middleton, Hewison, & Mueller, 1998; Roadhouse et al., 2018).

However, the diversity of views expressed and the sheer heterogeneity of lives affected by genetic disease—not only across diagnostic boundaries, but also within them (Boardman, Young, & Griffiths, 2016)—suggest that further research is required to better understand the perspectives of families and adults living with genetic disease and the associated factors that lend themselves toward either the support or nonsupport of genetic intervention in reproduction.

Drawing on a previous study of the views of families living with the neuromuscular disorder Spinal Muscular Atrophy (see Boardman et al., 2016), this exploratory analysis examines the reproductive views, decisions, and attitudes of families and adults living with hemophilia. It is only through such an understanding that the full range of possible impacts of genetic carrier screening for conditions such as hemophilia can be anticipated, and their relevance to the implementation of future programmes considered.

### 1.1 Hemophilia

Hemophilia is a rare, X‐linked blood clotting disorder typically affecting males (with female carriers) that causes excessive and prolonged bleeding. In some circumstances, females can be affected by hemophilia if both of their parents are homozygous for the mutation (i.e., they carry the same mutation), however this is considered very rare. Children born with hemophilia today can reasonably expect a normal or near‐normal lifespan (Haemophilia Society, 2003), although this varies significantly across the developing world (Ghosh, Shetty, Pawar, & Mohanty, 2002). Hemophilia A is the most severe and most common form of the disorder, accounting for around 70% of all diagnoses (Haemophilia Society, 2003). People with hemophilia A are particularly vulnerable to injury and can experience spontaneous internal bleeds (often into joints) leading to acute pain and long‐term disability. The most common cause of hemophilia‐related deaths are due to intracranial bleeds.

Hemophilia B is generally understood to be a less severe form of the disease, but for individuals with hemophilia B, spontaneous and internal bleeds can affect daily living. Treatment focuses primarily on replacing lost blood plasma and the administration of recombinant concentrates to substitute the absent clotting factor. More recently, the introduction of prophylactic IV therapies have been proven effective at preventing bleeds in those with hemophilia A. The administration of contaminated blood products to people with hemophilia throughout the 1980s, however, has also led to the development of substantial co‐morbidities in older men with hemophilia (typically hepatitis B/C and HIV), as well as increased social stigma for many people living with hemophilia in the UK (Barlow, Stapley, & Ellard, 2007).

### 1.2 Genetic testing for hemophilia

Prenatal genetic diagnosis for pregnant women already known to be carriers of hemophilia became available during the 1970s using analysis of fetal blood at 18–20 weeks’ gestation (Mårtensson, Tedgård, & Ljung, 2014), and pre‐implantation genetic diagnosis (which involves the creation of embryos through IVF and then testing them for hemophilia prior to implantation) became available to hemophilia carriers in the UK in 2004. Both of these forms of testing, however, are only available to families already at risk of having a child with hemophilia (not the general population), and typically only those living with “severe” forms of the condition (Hill, Compton, Lewis, Skirton, & Chitty, 2012). Furthermore, the recent introduction of NIPT (Non‐Invasive Prenatal Testing) within NHS healthcare, is also likely to contribute to the declining use of invasive prenatal testing for hemophilia. As NIPT can now be used to accurately determine fetal sex early in pregnancy, the unnecessary testing of female fetuses is gradually being eliminated (Hill et al., 2012).

### 1.3 Testing and screening: The views of families and adults with hemophilia

Despite hemophilia being the most common inherited blood disorder, no previous studies exist that explore the views of both people with hemophilia and their families toward population carrier screening. This is in spite of the increasing recognition of the health implications of hemophilia carrier status (Hooper, Miller, & Key, 2010), and the rising healthcare costs associated with its management as more sophisticated treatments are developed, and adults with hemophilia live increasingly long lifespans (Tsai et al., 2013). Where the views of the general public toward hemophilia screening have been considered, however, strong support for screening, and a greater willingness (than hemophilia carriers) to terminate a pregnancy affected by hemophilia have been observed, although there remains a notable lack of evidence in this area (Tsai et al., 2013, p. 724).

Indeed, the majority of previous research studies that have been conducted on uses of, and attitudes toward, genetic testing for hemophilia have all tended to focus on their use within already affected families, rather than by the general population. This research has shown that whilst there is generally great enthusiasm for the carrier testing of female relatives (often at young ages) (Dunn, Miller, Griffioen, & Lee, 2008; Thomas et al., 2007), that attitudes toward pregnancy termination for hemophilia produce far more mixed results (Varekamp et al., 1990; Varekamp, Suurmeuer, Rosendaal, & Brocker‐Vriends, 1993). Indeed, a survey of 207 obligate and potential carriers in the Netherlands by Balak et al. (2012) suggested that there is broad support for the use of prenatal diagnosis (54% of their sample had accessed it) with 82% of identified affected pregnancies being terminated. However, evidence from other surveys of carriers contradict Balak et al.'s (2012) findings, with reports of far lower levels of acceptance of both prenatal testing, but also pregnancy termination (e.g., Kadir et al., 2000; Mårtensson et al., 2014; Dunn et al., 2008; Tsai et al., 2013; Varekamp et al., 1990; Ratna, Lehesjoki, Peippo, & Kaariainen, 1994; Kraus & Brettler, 1988). Varekamp et al.'s (1990) Dutch study, for example, revealed that only 31% of the 549 female carriers they surveyed would consider prenatal testing and selective termination for hemophilia, whilst Kadir's more recent (2000) UK‐based study of 197 female carriers produced a similar result, showing that only 27% of affected pregnancies (identified through prenatal testing) were terminated on the grounds of hemophilia (in spite of broad support for diagnostic testing). For surveys conducted by Kraus and Brettler (1988) and Ratna et al (1994), these figures were even lower, at 17% and 16% acceptance of testing and termination amongst female carriers respectively.

Key reasons for supporting and accessing prenatal diagnosis and selective termination for hemophilia have been identified as relating to the severity of the hemophilia in the family, liberal attitudes toward pregnancy termination and being of older age (Balak et al., 2012). Key reasons for non‐support/non‐use of prenatal testing and selective termination, however, include the acceptance of hemophilia as a “liveable” condition compatible with a good quality of life, fear of fetal loss associated with an invasive test, as well as religious objections to the interruption of pregnancy (Balak et al., 2012; Kadir et al., 2000; Kraus & Brettler, 1988; Ratna et al., 1994; Varekamp et al., 1990).

It is noteworthy, however, that such surveys have tended to focus exclusively on the views and decisions of female hemophilia carriers, to the exclusion of adult males with hemophilia themselves. Whilst this exclusion may be justified on grounds that it is carrier women (not men with hemophilia) to whom the technologies are offered, there are nevertheless compelling reasons to consider the views of genetically disabled adults in such debates, particularly given the range of possible negative impacts for them if mass screening were to be introduced. Where men with hemophilia have been included in research studies on this topic, there is some (limited) evidence to suggest that they hold more favorable attitudes toward hemophilia prevention than any other stakeholder group (Thomas et al., 2007, p. 637), however evidence on this remains scant.

To address this identified gap in the literature, this paper presents a mixed methods sequential analysis (Creswell & Plano Clark, 2006) of 22 in‐depth qualitative interviews and a national UK survey (*n* = 327) conducted with families and adults affected by hemophilia. The study explores their attitudes toward, and actual/anticipated uses of, prenatal testing, carrier testing, and selective pregnancy termination for hemophilia, but also their perceptions of the expansion of genetic screening for hemophilia beyond affected families to the general population. In so‐doing, this paper offers a contribution to the growing body of literature exploring the impacts of genomic medicine for those already living with genetic disease.

## 2 METHODS

### 2.1 Ethical compliance

Ethical approval was granted from the Biomedical and Scientific Research Ethics Committee for the qualitative interviews on 21/02/17 (REGO‐2017‐1910), and for the Hemophilia Screening Survey (UK) on 17/11/17 (REGO‐2017‐1910 AM02).

### 2.2 Data collection methods

This study involved the collection of both qualitative and quantitative data using an exploratory sequential mixed methods design. The data were collected between April 2017 and June 2018 through the use of exploratory in‐depth interviews (phase I), a quantitative survey (phase II), and an integrated mixed methods analysis (phase III).

#### 2.2.1 Qualitative interviews: Phase I

In order to explore the perspectives of families and adults living with hemophilia on screening and reproductive genetics, a call for participants was placed in the Haemophilia Society's newsletters, website, and social media accounts. People with different types of bleeding disorder were initially included in this exploratory phase of the research. This strategy led to the successful recruitment of 22 participants (15 females and 7 males), with interviews taking place between April 2017 and March 2018 (see Table 1 for a breakdown of qualitative interview participants). Ten of the participants had a bleeding disorder themselves (six males with hemophilia, one male with factor XII deficiency, and three females with Von Willebrands disease), and participants were geographically dispersed throughout the UK. Given this dispersion, participants were given a choice of interview method: telephone, face‐to‐face, or email. This led to four interviews taking place face‐to‐face (three in the participant's home and one at the University) and 18 interviews were conducted by telephone. The average length of interview (for both face‐to‐face and telephone interviews combined) was 47 min.

**Table 1:** Qualitative interview participant characteristics and interview type

Participants	Numbers	Gender		Interview type	
		Female	Male	Face‐to‐face	Telephone
Diagnosed with hemophilia/VWB	10	3	8	2	8
Family member of person diagnosed with hemophilia	12	11	0	2	10
Totals	22	14	8	4	18

Within interviews, participants were asked about their experiences of living with hemophilia, its impact on daily life, their views on reproduction in the context of genetic technologies, as well as their perceptions of the possibility of population carrier screening for hemophilia. The interviews were all recorded and transcribed verbatim before being transferred to Nvivo 11 qualitative data analysis software for analysis. A constructivist approach to grounded theory data analysis was used which involved initial “open coding” before more hierarchical coding. A process of coding, refinement of concepts through analysis meetings and reference to the literature, followed by re‐coding was carried out over a period of three months until “data saturation” (i.e., no new themes were emerging) had occurred (Glaser & Strauss, 1967).

#### 2.2.2 Hemophilia Screening Survey (UK): Phase II

Following analysis of the qualitative data, and in reference to a previously developed survey (Boardman et al., 2016), the Haemophilia Screening Survey (UK) was developed in order to measure the prevalence of ideas about screening expressed in the qualitative interviews on a larger scale. Retaining the same basic structure of a previously administered survey, the SMA Screening Survey (UK) (Boardman et al., 2016), this new survey was tailored to the ideas expressed in the qualitative interviews, as well as factors unique to hemophilia, such as questions about treatment and exposure to contaminated blood. Questions designed to capture demographic information (such as educational attainment, religious faith, and ethnicity) were either directly replicated from, or appear as modified versions of, questions included in the 2011 UK Census survey. Within the survey, participants were asked about their support for two different types of screening programme: Preconception genetic screening (PCGS), a programme to identify carriers of hemophilia before a pregnancy is established and prenatal genetic screening (PNGS) to identify carriers (and potentially also affected foetuses) during pregnancy.

As well as the initial qualitative work, the survey was passed through an expert panel for feedback and cognitive interviewing before distribution. Cognitive interviewing is a widely used technique that uses in‐depth interviewing techniques in order to explore the mental processes that participants use to answer survey questions (Willis, 2005). The expert panel was made up of three people living with hemophilia in their family, two staff members of the UK Haemophilia Society and a hemophilia specialist nurse.

Survey data collection was carried out over a period of six months, between January and June 2018. Participants were invited to complete it if they were aged over 18 and either had hemophilia themselves or had at least one diagnosis of hemophilia in their family. No restrictions were placed on the nature of the familial relationship, for example, step, adopted and fostered family members were all included as the social relationship to the person with haemophilia was considered as important as the biological relationship in determining screening attitudes. Including various family members in the study also enabled an examination of the effect on reproductive attitudes that differing levels of proximity to hemophilia had.

A paper version of the survey was mailed to all households known to the Haemophilia Society UK, and an online version was also made available and distributed through the Haemophilia Society's online networks. A link to the survey was also made available through the research project's website and Twitter/Facebook feeds. Participants were encouraged to distribute the survey to interested family/friends affected by hemophilia, and postal survey returns were all processed using data scanning technology to reduce human error.

### 2.3 Survey data stratification and statistical analysis

Responses to each question were stratified as follows: gender (Male 1 vs. Female 0); age (35‐45 1 vs. other 0); qualifications (degree or above (1) vs. other (0)); religious (yes (1) vs. no (0)); do you have children (yes (1) vs. no (0)); relationship to hemophilia (adults with disease (AWD) (1) vs. family (0)); type of hemophilia associated with your family (A (1) or B (0)). For all questions regarding screening answers were stratified as either agree/strongly agree (1) or other (0). This was done because it allowed the simplest way of assessing the positive views of respondents.

The attitudes of families and adults with hemophilia toward PCGS and PNGS were compared to determine if there were any statistical differences. The following sub‐group analyses were performed: All responders were analyzed collectively to identify any overriding trends (all responders). Responses from families (all) and AwD (all) were compared to determine if living the disease altered. Sub‐analyses on responders associated with hemophilia A and B were then performed: (a) responses from families associated with hemophilia A were compared with responses from families with hemophilia B; (b) responses from adults with hemophilia A were compared with responses from adults with hemophilia B; (c) responses from families associated with hemophilia A were compared with responses from adults with hemophilia A; and (d) responses from families associated with hemophilia B were compared with responses from adults with hemophilia B.

In each of the sub‐group analyses, the individual questions were assessed and then responses correlated against support for screening. For each question the number of “agree” versus “other” responses were reported and statistical differences between the subgroups were assessed using a chi‐squared analysis (Graphpad Prism software, v6).

#### 2.3.1 Integrated mixed methods analysis: Phase III

Following the qualitative and quantitative data analysis, the qualitative data were returned to in order to further interrogate the findings. Statistically significant findings from the survey were cross‐referenced with related themes from the qualitative dataset to explore possible reasons for the finding. For example, the finding that the majority of the survey participants agreed that hemophilia can be compatible with a good quality of life was interpreted through the qualitative data surrounding day‐to‐day living with hemophilia. This technique of returning to the qualitative dataset following quantitative analysis is particularly well suited to the identification of contradictions and nuances within the data (Creswell & Plano Clark, 2006), as well as to offer more in‐depth explanations for unusual or unexpected findings. For a topic area as complex as screening, this technique proved particularly useful in both explaining and illustrating the key findings of the project. Qualitative excerpts chosen for inclusion in this paper were selected from the analysis of both phases I and III, and on the basis that they particularly eloquently or clearly reflect a theme that was significant to the overall analysis.

## 3 RESULTS

In total, 22 participants took part in an in‐depth qualitative interview, and 327 returned the Haemophilia Screening Survey (UK).

### 3.1 Quantitative data

#### 3.1.1 Cohort descriptive characteristics

Of the 327 participants, 148 were family members of people with hemophilia (75.7%) and 179 had hemophilia themselves (24.3%; Table 2). A total of 173/327 (53%) participants were male, 85% of whom had hemophilia (Table 2). The majority of participants were over the age of 35 (85%), were not educated to degree level (57%), were religious (56%), and had children (77%) (Table 2). Most participants were associate with hemophilia A 271/327 (83%) rather than hemophilia B. Eighty‐six per cent of family members (127/148) (86%) were associated with type A and 146/179 (82%) of adults with hemophilia had been diagnosed with this form of the condition (Table 2).

**Table 2:** Characteristics and demographics of survey responders. Demographics are shown for all responders (*n* = 327), responders associated with hemophilia families (families; *n* = 148), and individuals with hemophilia (adults with disease; *n* = 179)

Characteristic	All responders (*n* = 327)	Families (*n* = 148)	Adults with disease (*n* = 179)	*p*‐Value*
Gender—no. (%)				*<0.0001*
Male	173 (53%)	21 (14%)	152 (85%)	
Female	154 (47%)	127 (86%)	27 (15%)	
Age (years)				*0.0002*
18−25	11 (3%)	1 (1%)	10 (6%)	
26−34	38 (12%)	22 (15%)	16 (9%)	
35−45	68 (21%)	45 (30%)	23 (13%)	
46−55	60 (18%)	21 (14%)	39 (22%)	
56−65	65 (20%)	30 (20%)	35 (19%)	
>65	85 (26%)	29 (20%)	56 (31%)	
Qualifications				0.19
Degree or higher	142 (43%)	70 (47%)	72 (40%)	
Other/none	185 (57%)	78 (53%)	107 (60%)	
Religious				0.46
Yes	183 (56%)	88 (60%)	95 (53%)	
No	130 (40%)	55 (37%)	75 (42%)	
Prefer not to say	14 (4%)	5 (3%)	9 (5%)	
Parents				*<0.0001*
Yes	253 (77%)	135 (91%)	118 (66%)	
No	73 (22%)	12 (8%)	61 (24%)	
Prefer not to say	1 (1%)	0		
Type of hemophilia				0.31
Hemophilia A	273 (83%)	127 (86%)	146 (82%)	
Hemophilia B	54 (17%)	21 (14%)	33 (18%)	

Notes

Response distributions were compared between families and adults with disease and significant differences were assessed using chi‐squared analysis (*p*‐value).

* Significant differences are in italics (*p* < 0.05).

#### 3.1.2 Preconception genetic screening

Overall**, **57% of survey participants were in favor of PCGS and there was no statistical difference in the levels of support between families and adults with disease (57% vs. 56%; *p* = 0.82) (Tables 3 and 4). The level of support was similar in all analyzed subgroups: (a) families associated with hemophilia A versus families associated with hemophilia B (57% vs. 62%, *p* = 0.65); (b) adults with hemophilia A versus adults with hemophilia B (56% vs. 55%, *p* = 0.86); (c) families associated with hemophilia A versus adults with hemophilia A (57% vs. 56%, *p* = 0.99); and (d) families associated with hemophilia B versus adults with hemophilia B (62% vs. 55%, *p* = 0.77) (Tables 3 and 4).

**Table 3:** Response summaries for questions assessing views on preconception genetic screening (PCGS). A) Response breakdowns are shown for family sub‐groups (all, hemophilia A and hemophilia B) and adults with disease sub‐groups (all, hemophilia A and hemophilia B)

Question	All Responders (*n* = 327)	*F* (all) (*n* = 148)	*F* (Hem A) (*n* = 127)	*F* (Hem B) (*n* = 21)	AwS (all) (*n* = 179)	AwD (Hem A) (*n* = 146)	AwD (Hem B) (*n* = 33AwD (Haem A) (*n* = 146)
Identifying carriers of hemophilia before a pregnancy is conceived will affect people's choice of reproductive partner (the person you choose to have a baby with)							
Agree	121 (37%)	52 (35%	46 (36%)	6 (29%)	69 (39%)	56 (38%)	13 (39%)
Other	206 (63%)	96 (65%)	81 (64%)	15 (71%)	110 (61%)	90 (62%)	20 (61%)
Identifying carriers of hemophilia in the general population will lead to carriers feeling stigmatized or different							
Agree	127 (39%)	54 (36%)	48 (38%)	6 (29%)	73 (41%)	65 (45%)	8 (24%)
Other	200 (61%)	94 (64%)	79 (62%)	15 (71%)	106 (59%)	81 (55%)	25 (76%)
Identifying carriers of hemophilia before a pregnancy is established is a good thing, as it will reduce the number of terminations due to hemophilia							
Agree	200 (61%)	93 (63%)	78 (61%)	15 (71%)	107 (60%)	89 (61%)	18 (55%)
Other	127 (39%)	55 (37%)	49 (39%)	6 (29%)	72 (40%)	57 (39%)	15 (45%)
Identifying carriers of hemophilia in the general population will increase awareness of Bleeding Disorder as a condition							
Agree	250 (76%)	113 (76%)	95 (75%)	18 (86%)	137 (77%)	111 (76%)	26 (79%)
Other	77 (24%)	35 (24%)	32 (25%)	3 (14%)	42 (23%)	35 (24%)	7 (21%)
People from the general population won’t be interested in finding out their carrier status for hemophilia as they won’t think its relevant to them							
Agree	175 (54%)	86 (58%)	73 (57%)	13 (62%)	89 (50%)	73 (50%)	16 (48%)
Other	152 (46%)	62 (42%)	54 (43%)	8 (38%)	90 (50%)	73 (50%)	17 (52%)
Preconception genetic screening is a form of “social engineering” (a way of controlling the genetic make‐up of the population)							
Agree	116 (35%)	47 (32%)	42 (33%)	5 (24%)	69 (39%)	54 (37%)	15 (45%)
Other	211 (65%)	101 (68%)	85 (67%)	16 (76%)	110 (61%)	92 (63%)	18 (55%)
I would support a preconception genetic screening programme for hemophilia							
Agree	185 (57%)	85 (57%)	72 (57%)	13 (62%)	100 (56%)	82 (56%)	18 (55%)
Other	142 (43%)	63 (43%)	55 (43%)	8 (38%)	79 (44%)	64 (44%)	15 (45%)

Note

Responses for each question were stratified as “agree” versus “other” (other = disagree and neither disagree nor agree)

**Table 4:** Response summaries for questions assessing views on preconception genetic screening (PCGS). A) Response breakdowns are shown for family sub‐groups (all, hemophilia A and hemophilia B) and adults with disease sub‐groups (all, hemophilia A and hemophilia B). Responses for each question were stratified as “agree” versus “other” (other=disagree and neither disagree nor agree)

Question	*F* vs. AwD (all)	*F* (Hem A vs. B)	AwD (Hem A vs. B)	Hem A (*F* vs. AwD)	Hem B (*F* vs. AwD)
	*p*‐Value				
Identifying carriers of hemophilia before a pregnancy is conceived will affect people's choice of reproductive partner (the person you choose to have a baby with)	0.56	0.49	0.91	0.81	0.56
Identifying carriers of hemophilia in the general population will lead to carriers feeling stigmatized or different	0.49	0.41	0.03	0.27	0.75
Identifying carriers of hemophilia before a pregnancy is established is a good thing, as it will reduce the number of terminations due to hemophilia	0.64	0.37	0.49	0.99	0.26
Identifying carriers of hemophilia in the general population will increase awareness of Bleeding Disorder as a condition	0.99	0.27	0.73	0.88	0.72
People from the general population won’t be interested in finding out their carrier status for hemophilia as they won’t think its relevant to them	0.14	0.71	0.87	0.22	0.41
Preconception genetic screening is a form of “social engineering” (a way of controlling the genetic make‐up of the population)	0.24	0.39	0.36	0.52	0.15
I would support a preconception genetic screening programme for hemophilia	0.82	0.65	0.86	0.99	0.77

The main reasons participants supported PCGS were because it would reduce the number of terminations (all: 61%, Families: 63%, AwD: 60%; Tables 3 and 4) and that it would raise awareness of hemophilia in the general population (all: 76%, Families: 76%, AwD: 77%; Tables 3 and 4). Again, there was no significant difference in responses between any of subgroups for these two questions (Tables 3 and 4).

Most of the responders did not agree that carrier screening and PCGS would alter people's choice in reproductive partner (all: 37%, Families: 35%, AwD: 39%; Tables 3 and 4) or that PCGS was a form of social engineering (all: 35%, Families: 32%, AwD: 39%; Tables 3 and 4). In addition, most participants did not believe carrier screening/PCGS would lead to stigmatization of carriers (all: 39%, Families: 36%, AwD: 41%; Tables 3 and 4); however, a higher percentage of adults with hemophilia A than adults with hemophilia B thought carrier stigmatization was an issue (45% vs. 24%, *p* = 0.03; Tables 3 and 4). This was the only significant difference identified between any of the subgroups for PCGS questions, which highlights the agreement between the different groups.

#### 3.1.3 Prenatal genetic screening

Overall, 59% of survey participants were in favor of PNGS and there was no statistical difference in the levels of support between families and adults with disease (60% vs. 58%; *p* = 0.73) (Tables 5 and 6). The level of support was similar in all analyzed subgroups: (a) families associated with hemophilia A versus families associated with hemophilia B (60% vs. 62%, *p* = 0.85); (b) adults with hemophilia A versus adults with hemophilia B (58% vs. 58%, *p* = 0.94); (c) families associated with hemophilia A versus adults with hemophilia A (60% vs. 58%, *p* = 0.81); and (d) families associated with hemophilia B versus adults with hemophilia B (62% vs. 58%, *p* = 0.78) (Tables 5 and 6).

**Table 5:** Response summaries for questions assessing views on prenatal genetic screening (PNGS)

Question	All responders (*n* = 327)	*F* (all) (*n* = 148)	*F* (Hem A) (*n* = 127)	*F* (Hem B) (*n* = 21)	AwS (all) (*n* = 179)	AwD (Hem A) (*n* = 146)	AwD (Hem B) (*n* = 33AwD (Hem A) (*n* = 146)
Identifying hemophilia in pregnancy will inevitably lead to less people with hemophilia coming into the world who could have lived fulfilling lives							
Agree	163 (50%)	71 (48%)	60 (47%)	11 (52%)	92 (51%)	75 (51%)	17 (52%)
Other	164 (50%)	77 (52%)	67 (53%)	10 (48%)	87 (49%)	71 (49%)	16 (48%)
Screening for hemophilia in pregnancy will enable everyone to make informed decisions about whether or not to bring a child with bleeding disorders into the world							
Agree	218 (67%)	91 (61%)	79 (62%)	12 (57%)	127 (71%)	104 (71%)	23 (70%)
Other	109 (33%)	57 (39%)	48 (38%)	9 (43%)	52 (29%)	42 (29%)	10 (30%)
Screening for hemophilia in pregnancy will prevent unnecessary suffering							
Agree	114 (35%)	48 (32%)	42 (33%)	6 (29%)	66 (37%)	53 (36%)	13 (39%)
Other	213 (65%)	100 (68%)	85 (67%)	15 (71%)	113 (63%)	93 (64%)	20 (61%)
Screening for hemophilia in pregnancy will raise awareness of the condition in the general population							
Agree	241 (74%)	108 (73%)	91 (72%)	17 (81%)	133 (74%)	109 (75%)	24 (73%)
Other	86 (26%)	40 (27%)	36 (28%)	4 (19%)	46 (26%)	37 (25%)	9 (27%)
It would be a loss to society to have less people with hemophilia coming into the world							
Agree	129 (39%)	66 (45%)	57 (45%)	9 (43%)	63 (35%)	54 (37%)	9 (27%)
Other	198 (61%)	82 (55%)	70 (55%)	12 (57%)	116 (65%)	92 (63%)	24 (73%)
It would be hard for pregnant women and their partners to refuse screening for hemophilia in pregnancy							
Agree	103 (31%)	48 (32%)	43 (34%)	5 (24%)	55 (31%)	47 (32%)	8 (24%)
Other	224 (69%)	100 (68%)	84 (66%)	16 (76%)	124 (69%)	99 (68%)	25 (76%)
Screening for hemophilia in pregnancy is still useful even if they can't tell you how severely affected the child would be							
Agree	210 (64%)	88 (59%)	76 (60%)	12 (57%)	122 (68%)	98 (67%)	24 (73%)
Other	117 (36%)	60 (41%)	51 (40%)	9 (43%)	57 (32%)	48 (33%) 9 (27%)	
Termination of pregnancies affected by hemophilia is unfortunately necessary if we are to make sure that the condition is eliminated							
Agree	33 (10%)	12 (8%)	10 (8%)	2 (10%)	21 (12%)	16 (11%)	5 (15%)
Other	294 (90%)	136 (92%)	117 (92%)	19 (90%)	158 (88%)	130 (89%)	28 (85%)
Prenatal screening for hemophilia is useful, but only insofar as it can aid and preparation for the birth of a boy with hamophilia, not for the consideration of termination (abortion)							
Agree	227 (69%) 227 (69%)	109 (74%)	92 (72%)	17 (81%)	118 (66%)	92 (63%)	26 (79%)
Other	100 (31%)	39 (26%)	35 (28%)	4(19%)	61 (34%)	54 (37%)	7 (21%)
I would support a prenatal screening programme for hemophilia							
Agree	193 (59%)	89 (60%)	76 (60%)	13 (62%)	104 (58%)	85 (58%)	19 (58%)
Other	134 (41%)	59 (40%)	51 (40%)	8 (38%)	75 (42%)	61 (42%)	14 (42%)

Note

A) Response breakdowns are shown for family sub‐groups (all, hemophilia A and hemophilia B) and adults with disease sub‐groups (all, hemophilia A and hemophilia. B) Responses for each question were stratified as “agree” versus “other” (other=disagree and neither disagree nor agree).

**Table 6:** Response summaries for questions assessing views on preconception genetic screening (PCGS).

Question	*F* vs. AwD (all)	*F* (Hem A vs. B)	AwD (Hem A vs. B)	Hem A (*F* vs. AwD)	Hem B (*F* vs. AwD)
	*p*‐Value				
Identifying hemophilia in pregnancy will inevitably lead to less people with hemophilia coming into the world who could have lived fulfilling lives	0.57	0.66	0.98	0.54	0.99
Screening for hemophilia in pregnancy will enable everyone to make informed decisions about whether or not to bring a child with bleeding disorders into the world	0.07*	0.65	0.86	0.12	0.39
Screening for hemophilia in pregnancy will prevent unnecessary suffering	0.68	0.68	0.73	0.61	0.56
Screening for hemophilia in pregnancy will raise awareness of the condition in the general population	0.81	0.37	0.81	0.58	0.53
It would be a loss to society to have less people with hemophilia coming into the world	0.08*	0.86	0.29	0.21	0.25
It would be hard for pregnant women and their partners to refuse screening for hemophilia in pregnancy	0.81	0.36	0.37	0.79	0.99
Screening for hemophilia in pregnancy is still useful even if they can't tell you how severely affected the child would be	0.11	0.81	0.53	0.25	0.25
Termination of pregnancies affected by hemophilia is unfortunately necessary if we are to make sure that the condition is eliminated	0.35	0.78	0.49	0.41	0.69
Prenatal screening for hemophilia is useful, but only insofar as it can aid and preparation for the birth of a boy with hemophilia, not for the consideration of termination (abortion)	0.14	0.41	0.08*	0.12	0.99
I would support a prenatal screening programme for hemophilia	0.73	0.85	0.94	0.81	0.78

Note

A) Response breakdowns are shown for family sub‐groups (all, hemophilia A and hemophilia B) and adults with disease sub‐groups (all, hemophilia A and hemophilia B). B) Response distributions were compared using chi‐squared analysis (*p*‐value; *p = 0.05‐0.1).

The main reasons participants supported PNGS were because it would allow everyone to make informed decisions about the pregnancy (all: 67%, Families: 61%, AwD: 71%; Tables 5 and 6) and that it would raise awareness of hemophilia in the general population (all: 74%, Families: 73%, AwD: 74%; Tables 5 and 6)**. **Although there was no significant difference between the sub‐groups for either of these questions, the difference between families (all) and adults with hemophilia (all) was approaching significance (61% vs. 71%, *p* = 0.07; Tables 5 and 6).

Most of the participants did *not* agree that PNGS would prevent unnecessary suffering (all: 35%, Families: 32%, AwD: 37%; Tables 5 and 6) or that it would be hard for people to refuse screening (all: 31%, Families: 32%, AwD: 31%; Tables 5 and 6). Interestingly, however, most participants did not think it would be a loss to society to have fewer people with hemophilia being born, although the difference between families (all) and adults with hemophilia (all) was approaching significance (all: 39%, Families: 45%, AwD: 35%; Families (all) vs. AwD (all), *p* = 0.08; Tables 5 and 6).

Screening is principally about providing information to expecting parents; what parents subsequently decide to do with this information is an individual decision. With this in mind, two questions were specifically set: (a) whether termination is unfortunately necessary to eliminate hemophilia; and (b) whether PNGS was important to prepare parents for the birth of an affected child. Results categorically identify that in this cohort, participants believe that PNGS is important for preparation (all: 69%, Families: 74%, AwD: 66%), with far fewer believing termination was necessary to eliminate the disease (all: 10%, Families: 8%, AwD: 12%) (Tables 5 and 6). Again, although there were no significant differences between the sub‐groups, it is important to note that fewer adults with hemophilia A thought PNGS was important to allow parents to prepare than hemophilia A families (63% vs. 72%, *p* = 0.08; Tables 5 and 6).

### 3.2 Qualitative data

Our quantitative data suggest that whilst hemophilia‐affected families and adults broadly support PCGS and PNGS, that support for pregnancy termination where hemophilia is detected was nevertheless strikingly low (Tables 3, 4, 5, 6). In order to further explore this finding, the qualitative data were returned to in phase III of the study in order to explore possible reasons for this seeming contradiction.

In total, 22 participants completed an in‐depth qualitative interview between April 2017 and March 2018. Ten of the participants had hemophilia themselves (three had von Willebrand's Disease and six had hemophilia and one factor XIII deficiency), with the remaining 12 being the female family members (daughters/mothers/sisters/wives/grandmothers/aunts) of someone with a hemophilia (see Table 7). Thirteen of the 22 participants (59% of the sample) had at least two diagnoses of hemophilia in their family, and out of the 10 individuals who had hemophilia themselves, four also had (or experienced the loss of) an affected relative, highlighting the relatively high degree of recurrence of hemophilia within families. The majority (68%) of the sample was female (*n* = 15), with all seven male participants having hemophilia themselves (no unaffected male family members responded). The age range of the sample was 26–83.

**Table 7:** Age, sex, and relationship to bleeding disorder of qualitative interview participants

Pseudonym	Age	Sex	Condition in Family	Parent?	Number of Children with a Bleeding Disorder	Nature of experience with Bleeding Disorder
Kathy	66	F	VWB and hemophilia	✓	0	Kathy has von Willebrand's Disease. Father had hemophilia. Grandmother to two boys with hemophilia
Rosie	38	F	Hemophilia	✓	1	Father and son have hemophilia
Kiara	26	F	Hemophilia	×	0	Sister of two brothers with hemophilia & sister of female carrier. Own carrier status unknown.
						
Sophie	45	F	Hemophilia	✓	2	Mother of two sons with hemophilia.
Karen	38	F	Hemophilia	✓	1	Son has hemophilia
Jenny	46	F	Hemophilia	✓	1	Brother and son have hemophilia
Megan	40	F	Hemophilia	✓	1	Brother and son have hemophilia
Ellie	23	F	VWB	×	0	Has Von Willebrand's Disease, as does mother
Charlotte	52	F	VWB	✓	1	Has Von Willebrand's Disease, as does daughter (Ellie)
Sarah	41	F	Hemophilia (severe)	✓	0	Husband has severe hemophilia, daughter is a carrier
Chloe	39	F	Hemophilia B (severe)	✓	1	Son diagnosed with severe hemophilia B
Harriet	37	F	Hemophilia A (severe)	✓	2	Nephew and two sons have severe hemophilia A
Tim	51	M	Hemophilia A (severe)	×	0	Has severe hemophilia A
Mark	46	M	Factor XIII deficiency	✓	0	Has Factor XIII deficiency
Valerie	64	F	Hemophilia (mild)	✓	0	Husband has mild hemophilia, two daughters are obligate carriers, four grandsons have hemophilia
John	83	M	Hemophilia (mild)	✓	0	Has mild hemophilia, two carrier daughters and four grandsons have hemophilia (husband of Valerie)
Emma	38	F	Hemophilia	✓	1	Father has hemophilia, son has hemophilia, and three nephews have hemophilia
Alex	57	M	Hemophilia	✓	0	Has hemophilia, brother had hemophilia but died from hepatitis infection
Dylan	21	M	Hemophilia	×	0	Hemophilia & brother
Arun	28	M	Hemophilia	×	0	Has hemophilia
Jo	35	F	Hemophilia B	×	0	Father has hemophilia B, nephew has hemophilia B
Michael	71	M	Hemophilia	✓	0	Has hemophilia

Within their interviews, participants were asked to talk about their stories with hemophilia, its impact on their day‐to‐day lives, as well as their hopes and expectations for the future. Fourteen of the participants reported being very familiar with the condition, having grown up around people with hemophilia (either because they had one themselves, or a member of their immediate family did), whereas the remaining eight participants first encountered hemophilia later in life, one through having a delayed onset of hemophilia (John) and the remainder through either having an affected son (Sophie; Karen; Jenny; Chloe; Harriet) or marrying a man diagnosed with hemophilia (two participants—Sarah; Valerie) (see Table 7). However, even for these lesser experienced participants, there was only one participant (Karen, whose son with hemophilia was only eight months of age at the time of interview) who did not have at least five years of prior experience of living with hemophilia in some capacity before their interview.

It has been suggested in the literature (Varekamp et al., 1990) that unlike the trend observed with other X‐linked disorders such as fragile X syndrome (Kay & Kingston, 2002), that proximity to, and amount of contact with hemophilia impacts attitudes toward the disorder, with increased contact correlating with more positive and accepting attitudes about having a child with hemophilia oneself (Thomas et al., 2007). This finding was borne out in our qualitative data, particularly among women who had grown up with hemophilia.

Rosie was 38 at the time of her interview, working part‐time in a management job and had two sons, Jacob and Harvey aged 4 and 6, respectively. Rosie was an obligate hemophilia carrier, being the daughter of Roy, diagnosed with hemophilia in early childhood, along with his brother Les. Rosie described her decision to refuse diagnostic testing during both of her pregnancies, believing that hemophilia is insufficient reason to terminate a pregnancy, and Harvey, her eldest child, was diagnosed with hemophilia shortly after birth. Given her family's history, Rosie reported feeling somewhat prepared for what life with hemophilia would be like;

I think we were really well prepared because, you know, we knew it was a 50% chance. I'd done a lot of reading to sort of inform myself of like where things are, you know, where the treatments are at now. So I was kind of sure in my own mind what was going to be going on. We've taken the view all along, this is not a life limiting condition, in either sense of the word, and you know, my dad, although his health has suffered has, you know, he's rally driving, he's done sea sailing he's, you know, this isn't something that makes you stop living your life, it will be a part of our boy….. but for Harvey, it's 5% of who he is. The fact that he's like really sensitive and can be really stroppy is a bigger part of his personality and who is than the fact that his blood's a bit dodgy.

By using her father and uncle's experiences as her point of reference, Rosie was able to view Harvey's hemophilia as something that her family—already well versed in dealing with the condition—would take in their stride. Indeed, by comparing current hemophilia treatments to the treatments Roy and Les received as children (which included the accidental transmission of Hepatitis C to them both in early adulthood), Rosie described hemophilia as being “much less of a big deal” now than it was when her father and uncle were young. Indeed, her insights into their early experiences of treatment likely generated and facilitated a more positive view of current hemophilia treatments than she may have had without this prior knowledge. It was due to this availability of prophylaxis therapy that, Harvey, although six, had yet to experience a significant bleed and Rosie described his avid participation in physical activities including Taekwondo and trampolining—activities that simply would have been too risky for him to participate in prior to the commencement of prophylaxis.

For Rosie, as well as the majority of the other 21 participants, this view of hemophilia as a condition that is not “life limiting” translated into conflicted and sometimes negative attitudes toward a population screening programme for it, particularly on account of its associated with selective pregnancy termination. Whilst unequivocally supporting a woman's right to make an informed choice about her own body and life, Rosie queried whether hemophilia was sufficient grounds for what she described as such a “drastic” intervention, and her views were further reinforced (along with those of four other participants) by her experiences of both the implicit and explicit pressures she reported experiencing at the hands of clinicians to at least consider genetic testing and possible termination during her pregnancies;

….the consultant was very keen for us to have an amniocentesis. What he couldn't do though was convince me of the benefit compared to the cost on that, because at no point … Harvey having haemophilia would in no way have led to us terminating the pregnancy and therefore we were struggling to find a benefit to offset that risk because you know, it was up to a 3% risk [of miscarriage]. Well you know, that's one in 30 and you know you wouldn't … I mean you wouldn't send a kid to school if you knew one of them in the class wasn't going to come home would you? You know, so there was no benefit for us, so I didn't know why, you know, there was no reason in my mind to take that test.

For Rosie, her unwillingness to terminate any affected pregnancy counter‐balanced any argument in favor of submitting to an invasive test that carried the risk of fetal loss. Indeed, when considering the possibility of a population level prenatal genetic screening programme for hemophilia, this dislocation of screening practices from the next steps in the process (namely prenatal diagnosis and potentially also pregnancy termination) underpinned the majority of participants’ views on screening. By separating screening from its association with selective abortion, participants instead justified support for the practice on the grounds of the physical and psychological preparation of the parents and the practical/clinical preparations necessary for the birth of a boy with hemophilia.

Unlike Rosie, Karen (aged 38) came to her interview with the least prior experience of hemophilia of all the participants in the qualitative phase. Her son, Benji, was eight months old at the time of interview and had been diagnosed with hemophilia just two months previously, following significant bleeds into his legs from routine childhood vaccinations. Whilst Karen was clear in her support of both preconception and prenatal screening for hemophilia, this was presented, rather than a way of preventing lives with hemophilia, but instead as a means of protecting and caring for both carrier mothers and boys with hemophilia.

Yeah, I certainly wouldn't terminate [for haemophilia] …. Obviously it's devastating because you'd never want that for your child, but there's great support out there and it can be managed pretty well and in time it will get easier. But if they're doing HIV screening at the point of midwifery booking in, I don't see why they can't do haemophilia screenings at that point. Because I was … I had to go on heparin [during pregnancy with Benji], a blood thinning drug and obviously had I been under the haematology team and had I been screened for it [haemophilia carrier status] they wouldn't have probably given me it [the heparin], or at least I'd have been very closely monitored. I don't know if that's caused any damage to him [Benji]. I'd like to think it hasn't and he was protected by my placenta, but he was on blood thinning and obviously he was born by C‐section, so there could have been massive complications. So that would have been really helpful…. So yes, I do think it's very important to have screening.

For Karen, rather than a means of eradicating hemophilia (a response to prenatal testing frequently reported in the literature), she viewed prenatal screening as a means to source information that could actually be used to protect and preserve lives affected by hemophilia. This interpretation of the potential uses of prenatal screening were widely reflective of the attitudes of other participants in the study, as well as being reflected in the quantitative data. A small sub‐set of participants, however, all of whom had hemophilia themselves, expressed more conflicted views about screening, on the one hand viewing screening as an important source of information, but also as potentially leading to impossible decisions that the general public may be ill‐equipped to make.

Tim was 51 years old at the time of his interview, and was diagnosed with hemophilia A in the early 1970s when he was three years old. Tim described the negative reaction of his family to the diagnosis, in particular the guilt, shame, and stigma experienced by his parents, which appeared to have been absorbed, at least in part, by Tim and his sister Anita, both of whom had made a commitment from an early age not to reproduce and risk passing on the condition. This view was reinforced when Tim contracted both Hepatitis B and C in the 1980s, and at the time of interview, Tim also believed he could be HIV positive. His decision never marry or have children, whilst ultimately contributing to the breakdown of Tim's relationship, was nevertheless one he described as standing by as an adult. For Tim, population screening for hemophilia is important, not only because it has the potential to prevent children being born with hemophilia, but also because it allows parents to avoid becoming what he termed “hemophilia parents”, a job which he thought not everyone is well suited to take on:

I would say … and this is looking back onto my parents as well—it's always much harder to be the parent of a haemophiliac than to be the haemophiliac themselves. As a parent you have to deal with all the guilt and the care of the child, whatever that may involve and you don't know…. And most people can't fully take on board what that means anyway. Like I said previously, no matter what you can say to people, until you physically experience it, you've got no sense of proportion or what it actually means in reality. You know, I have been asked questions by various people about what haemophilia is really like, you know, but we're all different, that's the problem, and how I react and manage my haemophilia is completely different to how someone else may do. So yeah it's [screening] giving people decisions, but not necessarily the tools to actually make them.

By highlighting both the importance, but also the limits of the information that can be gathered from a screening test for hemophilia, Tim's perspective was typical of some of the older men with hemophilia within the sample who had experienced both the physical harms, but also the intensely damaging social stigma of having receiving contaminated blood products in the 1980s and 90s, which for Tim, and other participants, had resulted in severe health complications, social stigma and bullying (Mark; John; Michael). For hemophiliac men such as Tim, Mark, John, and Michael (see Table 7) whose life experiences with hemophilia drew little resemblance to those of younger generations with hemophilia described by Rosie and Karen (largely due to the introduction of prophylaxis from a young age), the possibility of being able to predict life quality and experiences with hemophilia from the results of genetic tests was viewed as unrealistic, even as they supported the basic premise of screening and early identification of hemophilia.

## 4 DISCUSSION

This paper, to the best of our knowledge, is the largest and most in‐depth study of attitudes toward hemophilia screening and prevention amongst families and adults living with hemophilia ever to be conducted. Within a broader cultural and technological context whereby genomic technologies are becoming both more efficient and more accessible than ever before, and yet paradoxically people with genetic disorders such as hemophilia are simultaneously living longer and more fulfilling lives (Cassis et al., 2012) , the reflections of affected adults and their families will only become increasingly important. Indeed, as a society, we are facing significant decisions about which conditions are considered serious enough to warrant their prevention (or even gradual elimination) through population level genomic screening and, as such, the accounts of people living with genetic disease—the best “experts” on their condition are critical to these decisions (Asch & Wasserman, 2014; Korngiebel et al., 2016; Petersen, 2006; Roadhouse et al., 2018; Wertz & Knoppers, 2002).

Indeed, there are a range of potential impacts that population‐level genomic screening could have that are unique to affected families and adults, that warrant their inclusion as stakeholders in screening debates. Such impacts include the possible reduction in research funding or availability of treatments, reductions in peer‐to‐peer support as well as the potential for increased stigmatization as the public profile of the disorder shifts to a “preventable” disorder.

This study has revealed that families and adults affected by hemophilia are largely aware of the range of possible impacts for them should screening be introduced and these concerns were key to explaining why support for preconception and prenatal genetic screening were markedly lower among families and adults living with hemophilia than has been observed in relation to other genetic conditions, such as Spinal Muscular Atrophy (Boardman et al., 2016). Indeed, the transition of hemophilia from a condition that was fatal in childhood in the 1970s, to one that is largely treatable, is likely to have influenced families’ views on the necessity and value of screening for the disorder.

Alongside shifts in the treatability of hemophilia, there have been concomitant and significant changes in the way in which hemophilia families both access, and make use of, prenatal diagnosis in recent years. The widespread introduction of NIPT has made fetal sex identification both more reliable and accessible, and at earlier points in pregnancy than was previously possible (Hill et al., 2012). The introduction of this technology, by “filtering out” female fetuses who will never develop hemophilia, has played a pivotal role in the sharp decline of invasive diagnoses for pregnant carrier women. However, along with a decline in accessing prenatal diagnosis, it has also been observed that rates of pregnancy termination when hemophilia is detected in this way, have also been steadily declining, as families increasingly access the test in order to make the birth as safe as possible for a hemophiliac baby, rather than to terminate the pregnancy (Mårtensson et al., 2014).

This trend toward testing for information for preparation purposes rather than to avoid genetic disease was reflected clearly in both our qualitative and quantitative data on screening, and may explain why PNGS garnered slightly more support from the cohort than PCGS (59% vs. 57%), despite the closer alignment of PNGS with pregnancy terminations. Indeed, whilst previous research has demonstrated a widespread correlation between accessing prenatal diagnosis and intentions to end affected pregnancies, this study suggests that the reverse is true for families and adults living with hemophilia. Confirming the findings of other studies exploring uptake of screening and pregnancy termination amongst families affected by genetic disease (Ryan, Miedzybrodzka, Fraser, & Hall, 2003), the findings of this study suggest that rather than a means of eliminating the condition, PNGS was viewed primarily as a means of supporting, or even *preserving* the lives of boys with hemophilia and carrier women (e.g., Karen). Indeed, 69% of the sample agreed that PNGS should be used exclusively for information purposes, and 90% disagreed on principle with the use of pregnancy termination to prevent hemophilia. Indeed, it was the implicit and explicit links between PNGS and pregnancy termination that led participants with extensive prior knowledge of hemophilia to reject PNGS (e.g., Rosie).

Unlike previous studies of the views of families and adults with genetic disease toward reproductive genetics (Boardman et al., 2016; Maxwell et al., 2011; Raspberry & Skinner, 2011), this study also highlights the strong degree of agreement in reproductive attitudes between adults with hemophilia and their family members. Furthermore, this agreement was retained even across the different types and severities of hemophilia. Despite their very different ways of knowing and experiencing hemophilia, both families and adults were largely united, not only in their support of screening, but also their disapproval of its uses for termination purposes. This finding underscores the centrality of direct lived experience to reproductive views and decisions, and the importance of considering the experiential knowledge of family members as well as those diagnosed with genetic disease, particularly parents and siblings whose lives are often fundamentally altered, albeit in different ways, by a hemophilia diagnosis in the family (Cassis et al., 2012; von der Lippe, Frich, Harris, & Solbrække, 2017).

The only key area of divergence between participant groups related to perceptions of carrier status stigmatization, where participants diagnosed with hemophilia A were found to be more likely than any other group to perceive stigma around carrier status. Given that the majority of adults diagnosed with hemophilia A who participated in the survey reported either having been exposed to, or having contracted, a blood born virus (most commonly HIV or Hepatitis B/C) in the 1980s or 90s, it is likely that experiences of social stigma related to contaminated blood were transferred into attitudes toward carrier identification. In addition to the possibility of blood contamination, the relative severity of hemophilia A and associated treatments may have also contributed to these differences, with research highlighting that most men with hemophilia are reluctant to disclose their condition beyond close family and friends (Cassis et al., 2012).

Finally, despite the fact that the majority of the sample supported the underlying principles of both PCGS and PNGS, albeit insofar as they can provide information and aid preparation, it is noteworthy that most (61%) simultaneously also agreed that a decline in people born with hemophilia would *not* be a loss to society. The inherent tensions between these two value statements, a rejection of pregnancy terminations for hemophilia on the one hand, but also a belief that the continued existence of hemophilia does not contribute anything to society worth preserving on the other, may at first glance appear contradictory, but in fact strikes at the very heart of the key issue facing reproductive genomic medicine today; namely, the conflict between preventing diseases from coming into existence, and preventing *the people *who would have those diseases from coming into existence. It is this distinction that has informed much of the disability rights responses to prenatal screening, testing, and pregnancy termination and plagued the accounts of parents of genetically disabled children considering future pregnancies (Kelly, 2009). Through their responses to both the survey and in‐depth interviews, it appeared that families and adults with hemophilia were largely able to disentangle their views about hemophilia from their views about people with hemophilia, which contributed to these seeming contradictions.

Indeed, the relationship between genetic disorders and personal/social identity is emerging as an important factor in recent analyses of the responses of disabled people to advances in genetic medicine more widely (Asch & Wasserman, 2014) and is likely to only become more significant overtime. Technologies such as genome editing suggest a future in which it may be possible to focus ameliorative responses to genetic disease solely on the disease causing genetic variant, whilst preserving the life of the would‐have‐been disabled fetus. Future research may usefully explore this role of identity politics within the responses of genetically disabled adults and their families toward population level genetic screening, as well as the types of experience with genetic disease that lend themselves to support or nonsupport of screening and disease prevention, particularly as they relate to pregnancy termination.

### 4.1 Strengths and weaknesses

A key strength of this study is the depth and breadth of data collated. However, due to confidentiality and data protection constraints, no identifiable data were asked of individuals who participated in the Haemophilia Screening Survey (UK), including IP address (where the survey was completed online). This meant that there was no mechanism in place to prevent an individual completing multiple surveys. Moreover, there was no way to verify that the participant fitted the inclusion criteria. Participants were furthermore accessed through a national support group rather than hemotology clinics, which may have introduced bias. Moreover, given the relatively high incidence of hemophilia A, people with hemophilia B were under‐represented in the sample, although their numbers were broadly reflective of the prevalence of these sub‐types of hemophilia within the UK population.

## CONFLICTS OF INTEREST

The authors have no conflicts of interest to declare.

## ETHICAL STATEMENT

Ethical approval for this study was granted by the Biomedical and Scientific Research Ethics Committee for the qualitative interviews on 21/02/17 (REGO‐2017‐1910), and for the Haemophilia Screening Survey (UK) on 17/11/17 (REGO‐2017‐1910 AM02).
